# Supplementary material for: Cycloidal-spiral sampling for three-modal x-ray CT flyscans with two-dimensional phase sensitivity
Source: Sci Rep. 2022 Dec 9;12:21336. doi: 10.1038/s41598-022-25999-1 (PMC9734192; doi:10.1038/s41598-022-25999-1)
Supplement: Supplementary file 1 — Supplementary Information. [file 41598_2022_25999_MOESM1_ESM.docx]

**Supplementary material**


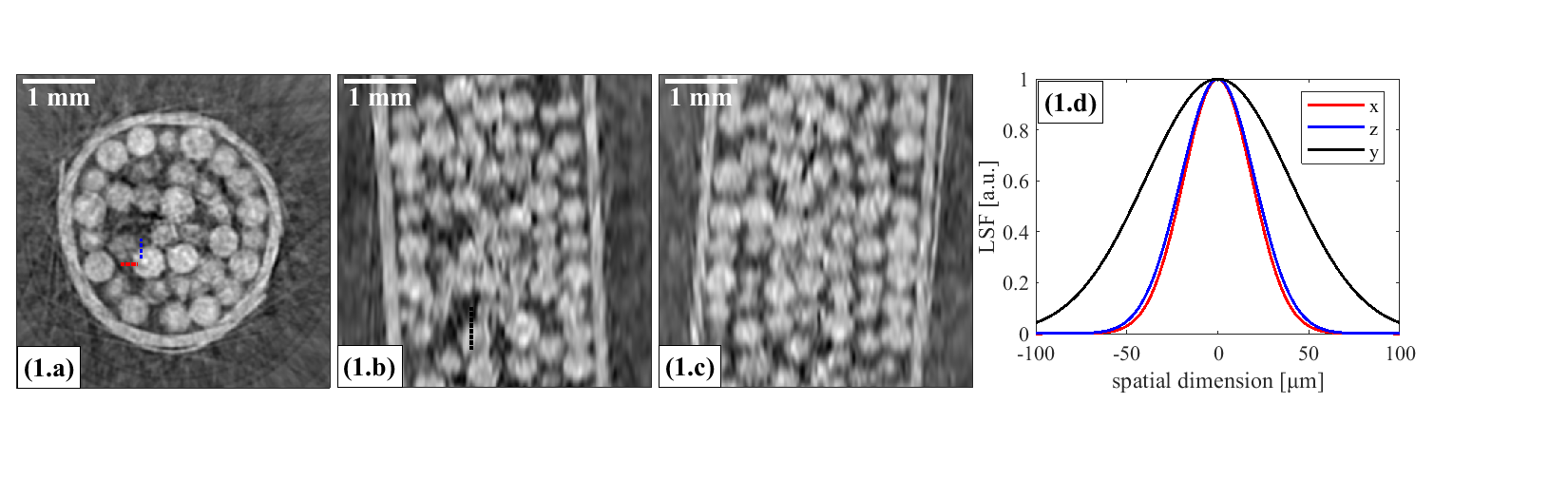

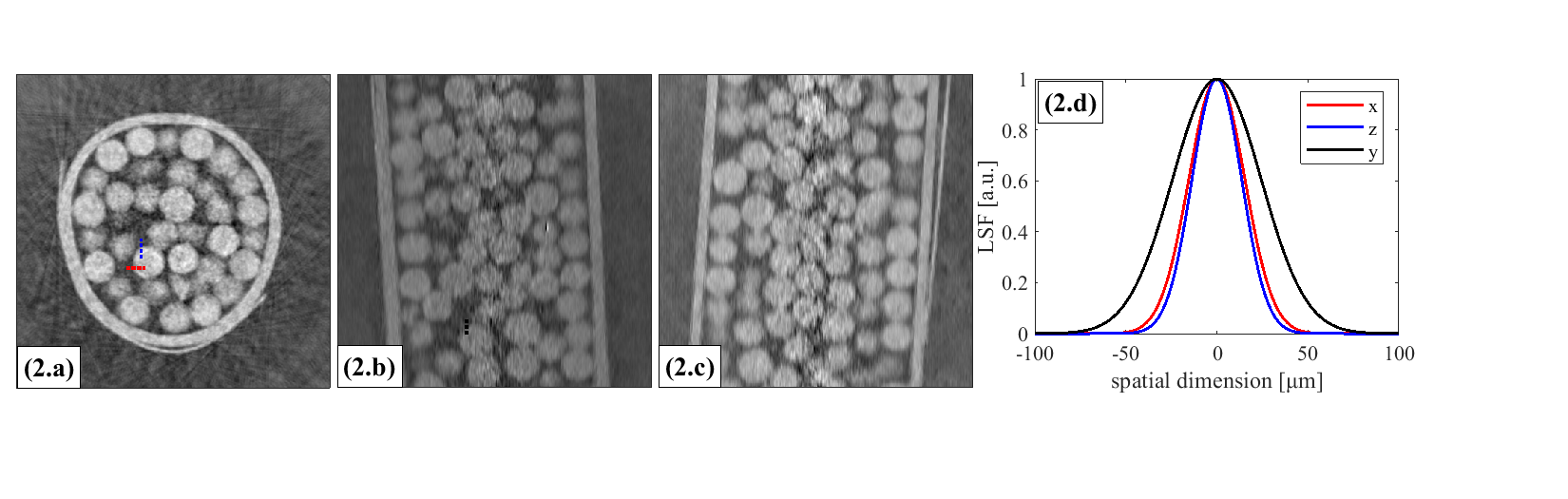

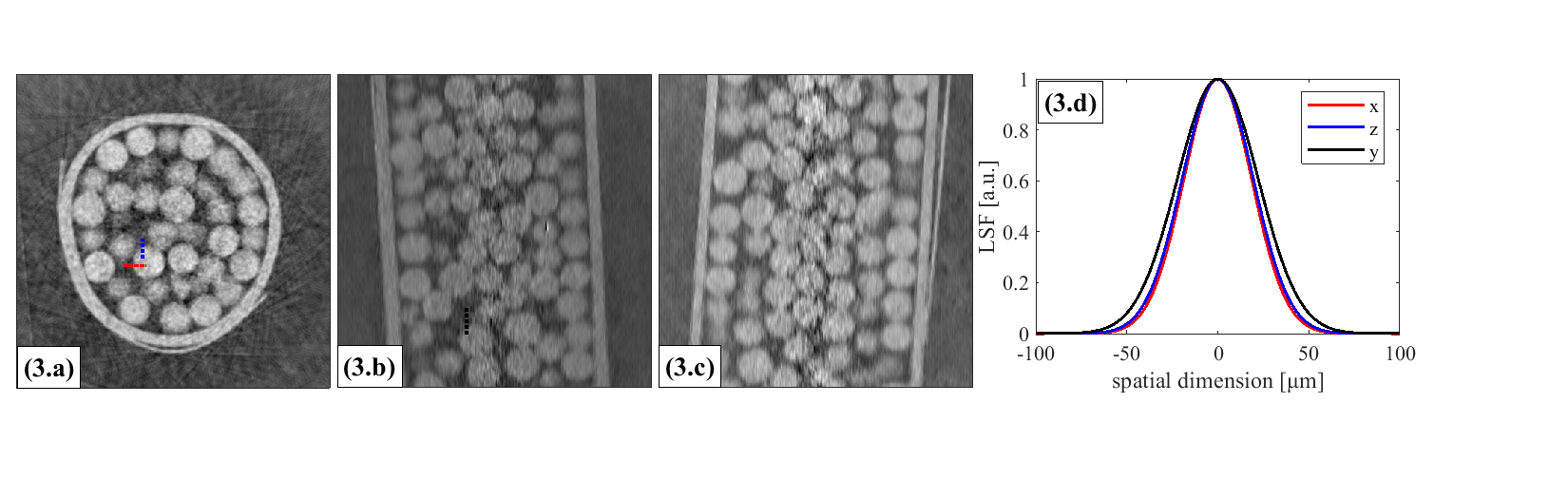

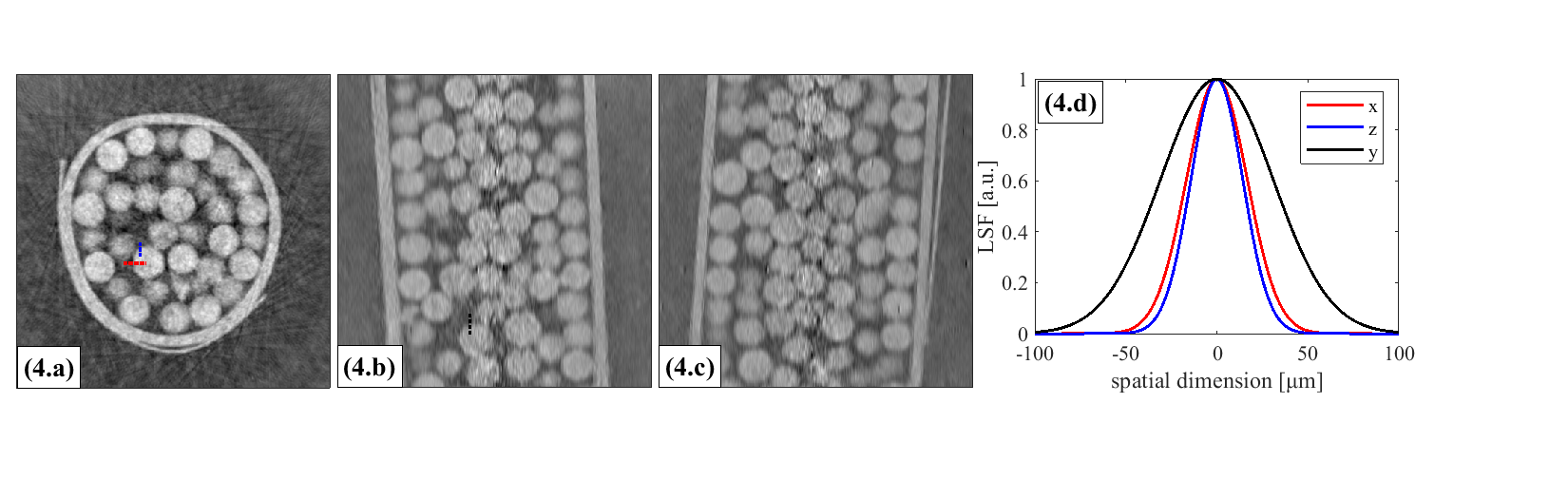

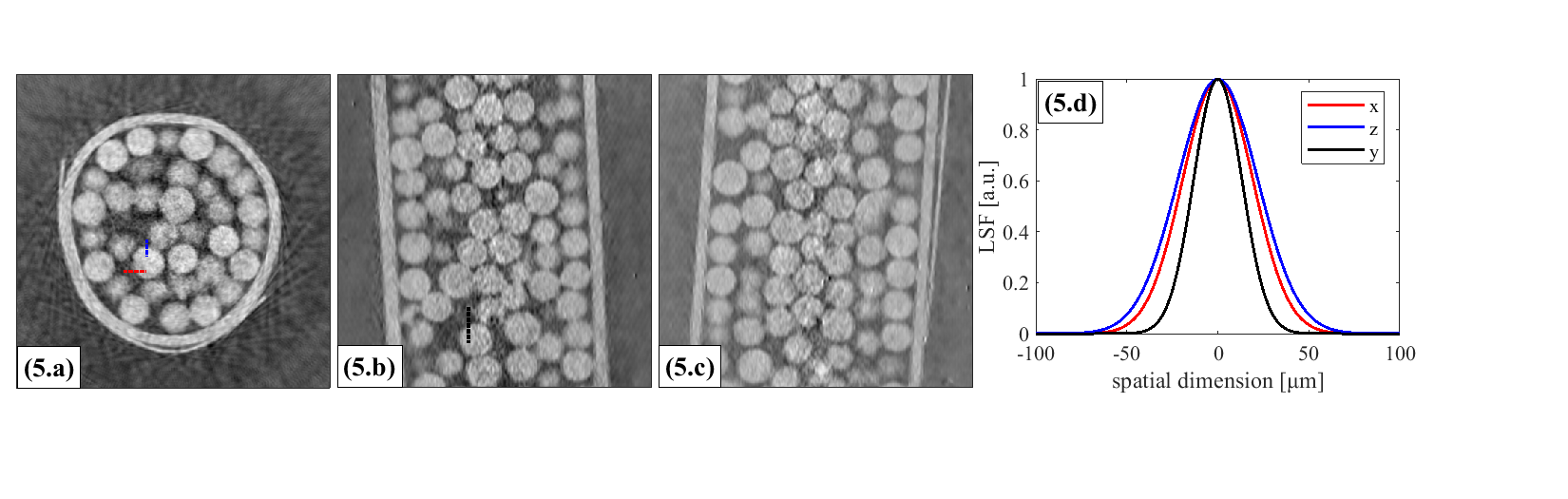

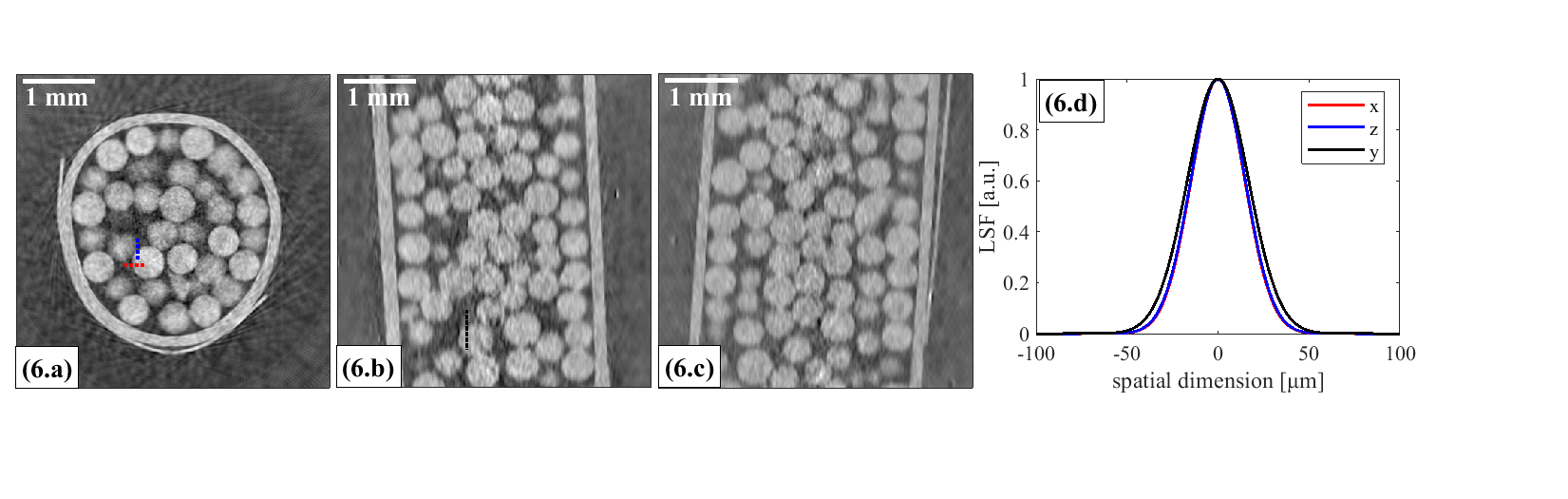

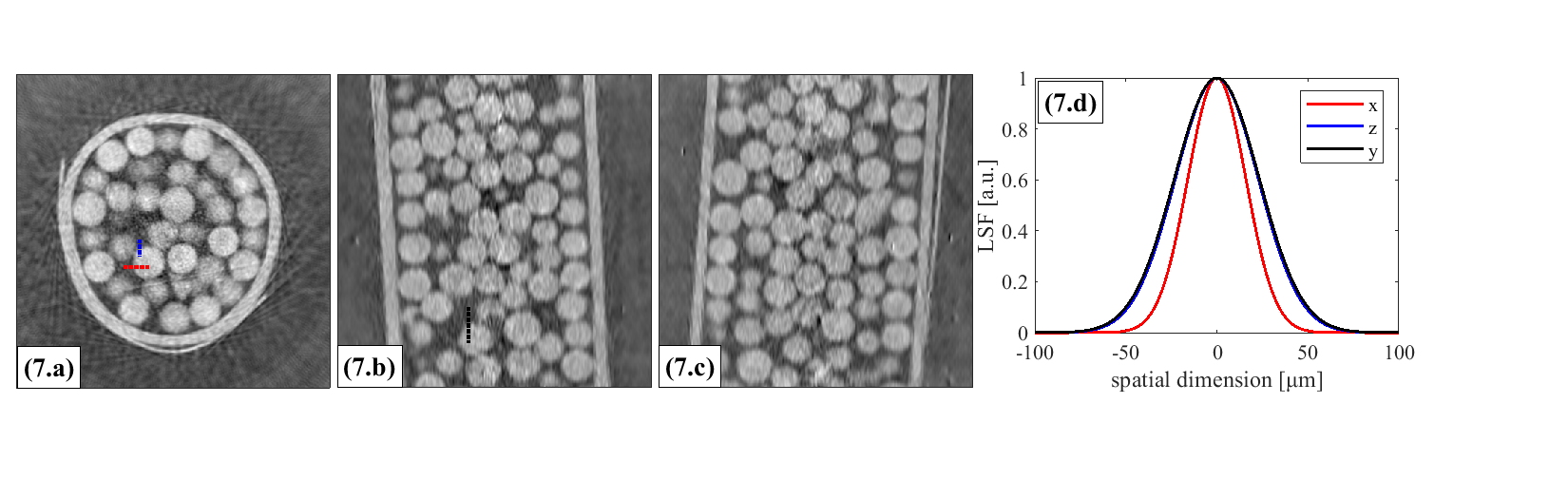

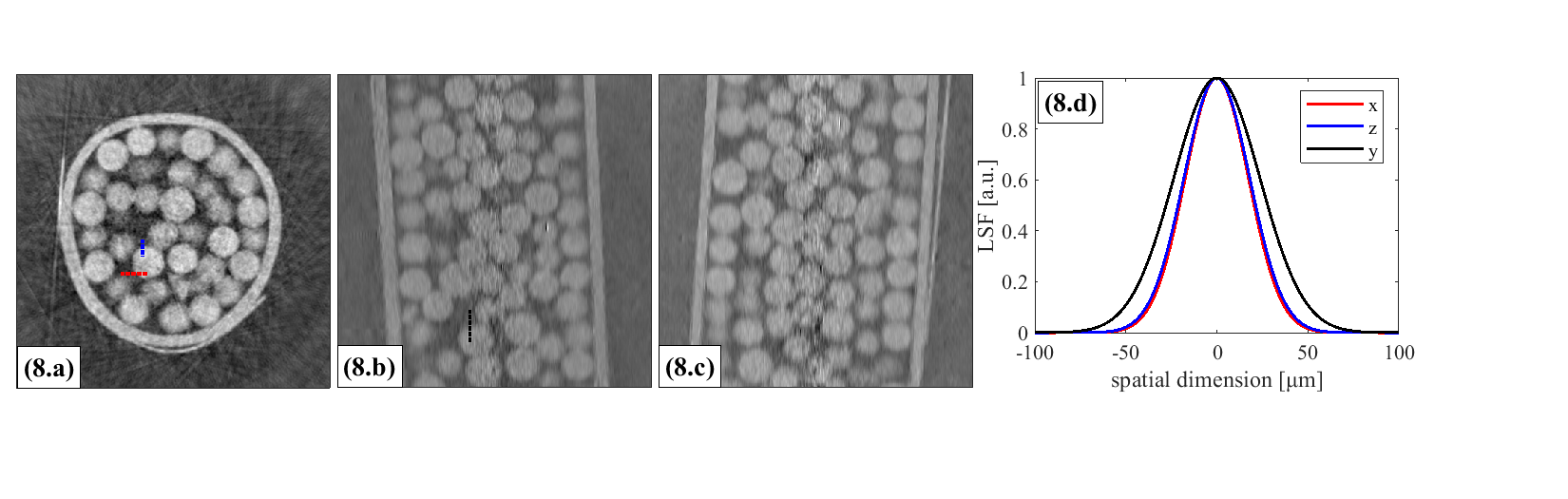

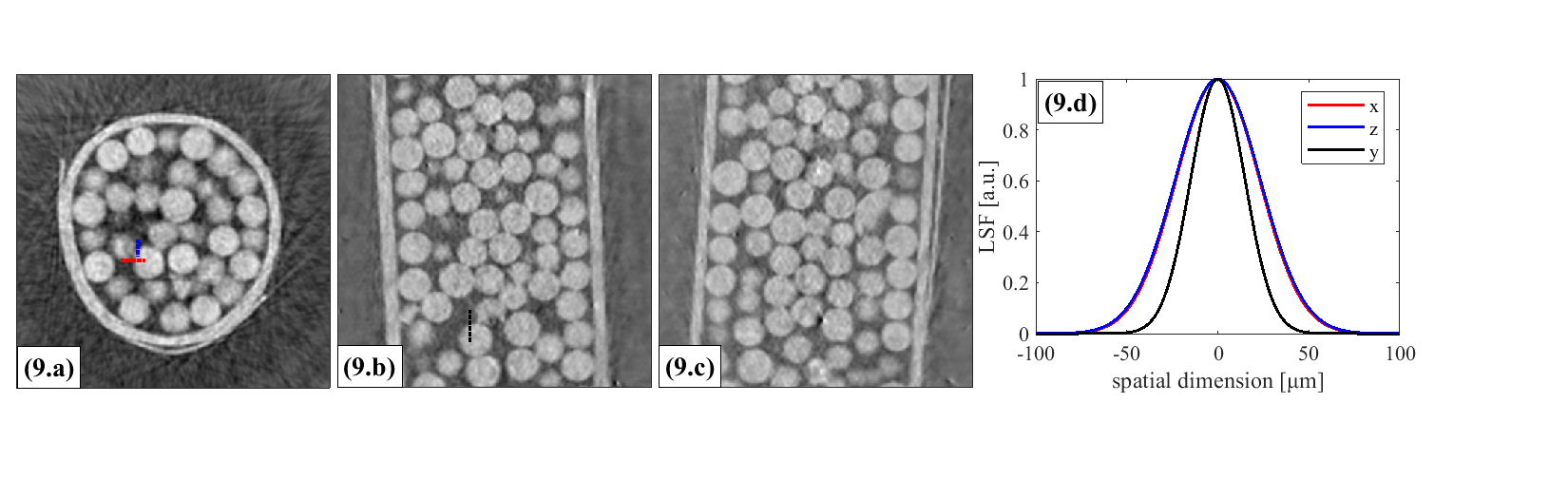

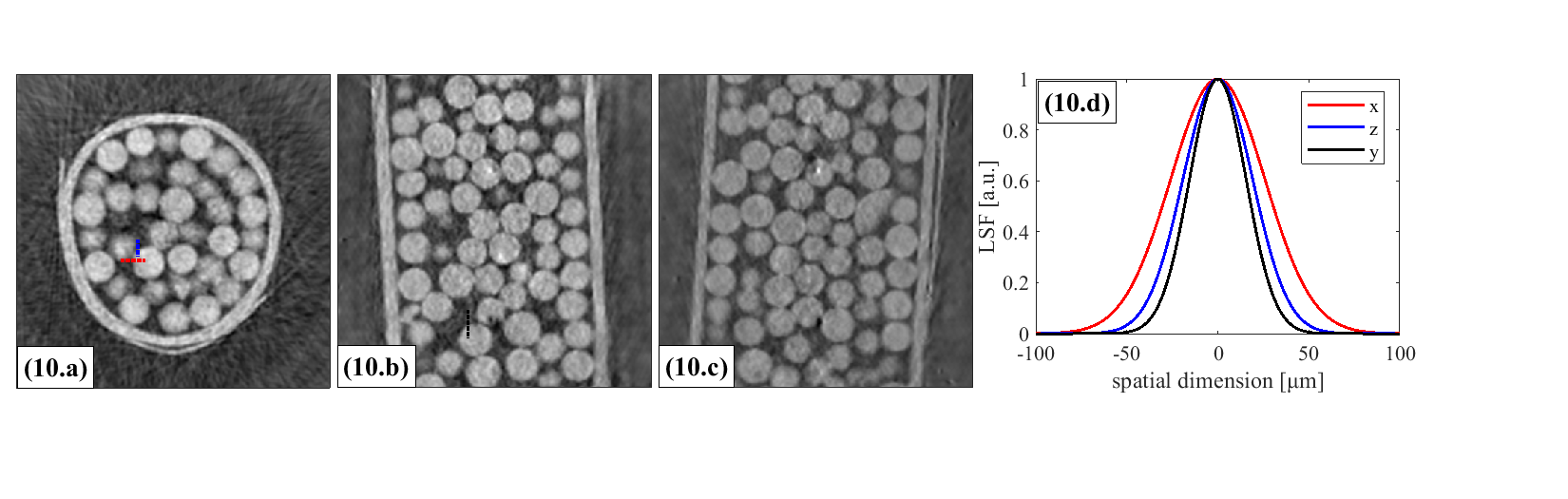

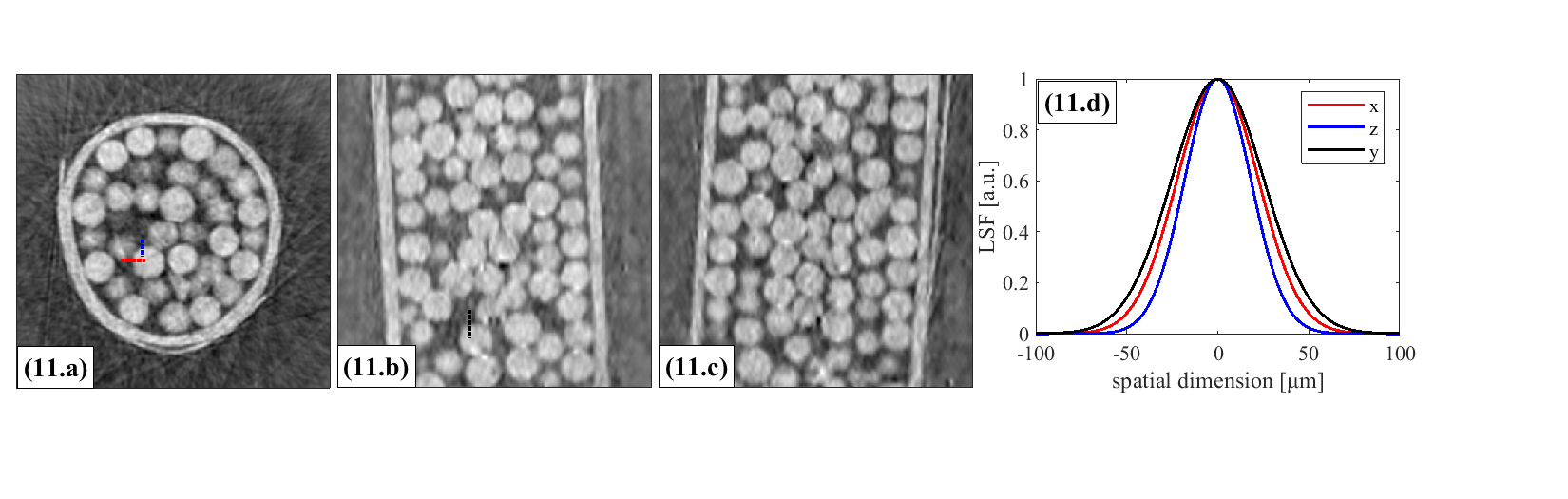


**Fig. SM1.** Reconstructed (a) axial, (b) sagittal, and (c) coronal planes of the spheres phantom; (d) the line spread functions along the *x* (red line), *y* (black line), and *z* (blue line) direction, as indicated in the images. Results are shown for sub-sampling the fully sampled (dithered) dataset according to a cycloidal-spiral scheme with (1) *dx*= 0 μm, *dy*= 0 μm (which in fact corresponds to a rotation-only scan), (2) *dx*= 10 μm, *dy*= 10 μm, (3) *dx*= 10 μm, *dy*= 20 μm, (4) *dx*= 10 μm, *dy*= 40 μm, (5) *dx*= 20 μm, *dy*= 10 μm, (6) *dx*= 20 μm, *dy*= 20 μm, (7) *dx*= 20 μm, *dy*= 40 μm, (8) *dx*= 30 μm, *dy*= 30 μm, (9) *dx*= 40 μm, *dy*= 10 μm, (10) *dx*= 40 μm, *dy*= 20 μm, (11) *dx*= 40 μm, *dy*= 40 μm.


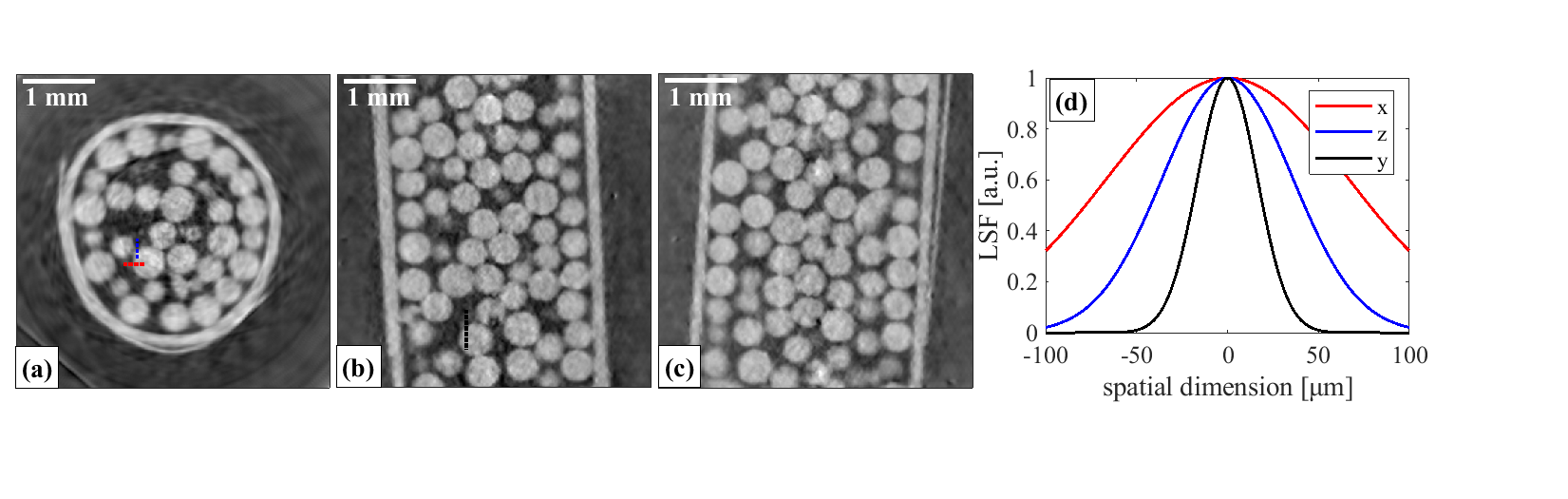

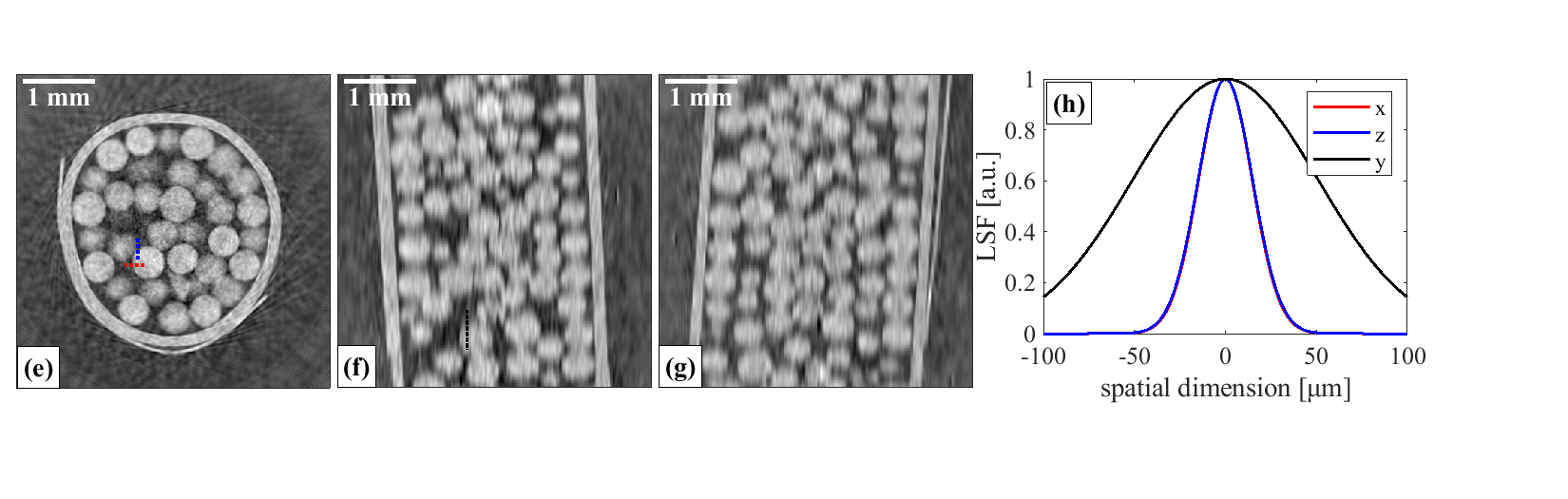

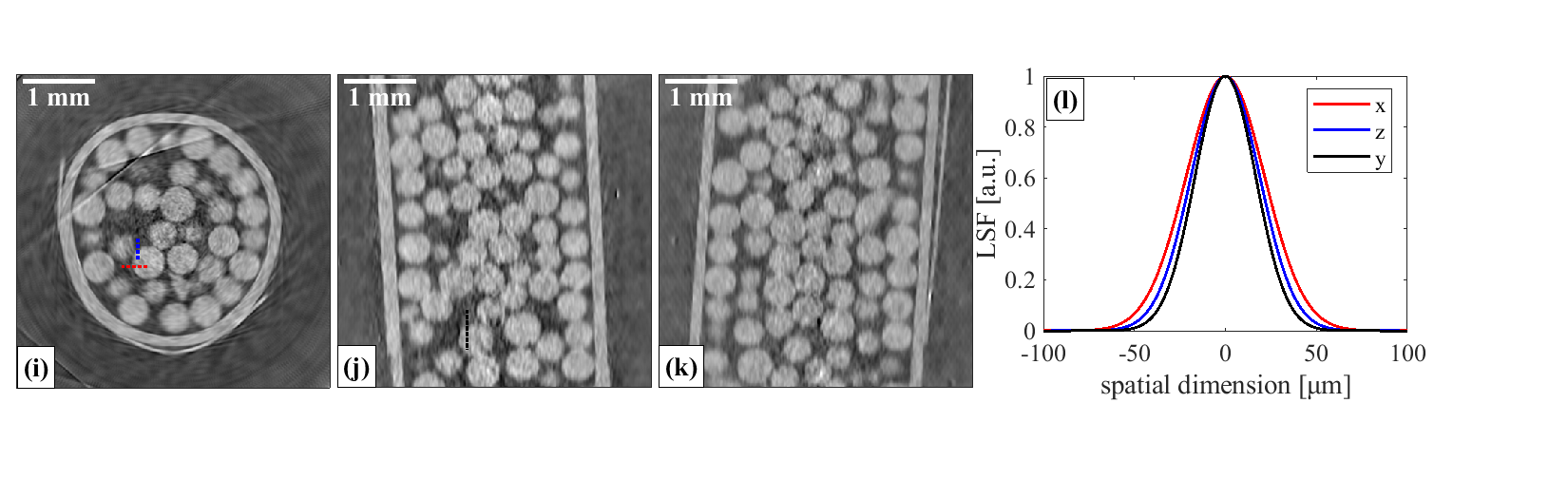


**Fig. SM2.** Reconstructed axial (left), sagittal (central left), and coronal (central right) planes of the sphere phantom, and the corresponding line spread functions (right) extracted from the sphere edges indicated with corresponding colours along the *x*, *y* and *z* axes. Results are shown for a cycloidal-spiral sub-sampling with *dx*= *dy*= 20 μm, with the missing entries in the 3D dataset interpolated with a 3D interpolation approach (a-d); the described 1D-2D interpolation approach (e-h); and the described 2D-2D interpolation approach (i-l).

**Figure SM2** shows results obtained by applying three different methods of interpolation (a fully 3D approach, the described 1D-2D approach, and the described 2D-2D approach; cubic interpolation for 1D and 2D, natural neighbor interpolation for 3D) to fill the missing entries of the subsampled cycloidal-spiral scan with *dx*= *dy*= 20 μm. The three methods were compared by visual inspection and based on spatial resolution estimates extracted from the slices. The resolution along *x*, *y*, *z* was estimated to be 160 μm ± 30 μm, 39 μm ± 10 μm, 85 μm ± 20 μm for the fully 3D approach, 35 μm ± 9 μm, 120 μm ± 20 μm, 35 μm ± 3 μm for 1D-2D approach, and 50 μm ± 30 μm, 39 μm ± 7 μm, 44 μm ± 4 μm for the 2D-2D approach. In addition to this, the computation time for each interpolation approach was considered; the 3D interpolation took 860 minutes, the two steps 1D-2D approach took 22 minutes, and the two steps 2D-2D approach took 38 minutes, for all five contrast channels (attenuation, two-dimensional refraction, and two-dimensional scattering) to be computed. On balance, we concluded that a combined 1D-2D / 2D-2D approach (whereby 1D-2D interpolation is used to reconstruct axial slices, and 2D-2D interpolation is used to reconstruct coronal and sagittal slices) was the best option for use in the context of this article.
